# TiO_2_ Decorated onto Three-Dimensional Carbonized Osmanthus Fragrans Leaves for Solar-Driven Clean Water Generation

**DOI:** 10.3390/nano15070504

**Published:** 2025-03-27

**Authors:** Yali Ao, Li Wang, Lin Yang, Chengjie Duan, Qizhe Gui, Songyun Cui, Shutang Yuan, Jiaqiang Wang

**Affiliations:** 1 School of Materials and Energy, Institute of International Rivers and Eco-Security, Yunnan Province Innovation Center for New Materials and Equipment Technology in Water Pollution Control, Yunnan Frontier Water Environment Industry Research Institute, Yunnan University, Kunming 650091, China; yl_a2019@163.com (Y.A.); li_wang2025@163.com (L.W.); 15687376526@163.com (L.Y.); duanchengjie@stu.ynu.edu.cn (C.D.); grant7@163.com (Q.G.); 2 Kunming Branch of Yunnan Hydrology and Water Resources Bureau, Dianchi Lake Ecosystem Observation and Research Station of Yunnan Province, Kunming 650032, China; csy126127@163.com (S.C.); kmxyjs@126.com (S.Y.)

**Keywords:** solar steam generation, TiO_2_ composites, carbonized biomass, water treatment

## Abstract

Solar steam generation (SSG) has garnered significant attention for its potential in water purification applications. While composites with physically combined structures based on semiconductors or biomass have been developed for SSG, there remains a critical need for low-cost, high-efficiency devices. In this study, TiO_2_ composites exhibiting excellent stability, high solar absorption, porous microstructure, and hydrophilic surfaces were identified as effective materials for SSG and water purification for the first time. A novel SSG device was designed by decorating TiO_2_ onto three-dimensional carbonized Osmanthus fragrans leaves (TiO_2_/carbonized OFL). Compared to directly carbonized OFL (without TiO_2_) and Osmanthus fragrans leaves with templated TiO_2_ (OFL-templated TiO_2_), the TiO_2_/carbonized OFL carbon composites demonstrated enhanced solar absorption, achieving over 99% in the visible region and more than 80% in the near-infrared region. Under solar illumination of 1 kW·m^−2^, the TiO_2_/carbonized OFL device achieved a high water evaporation rate of 2.31 kg·m^−2^·h^−1^, which is 1.6 times higher than that of carbonized OFL and 3.45 times higher than OFL-templated TiO_2_. Additionally, the TiO_2_/carbonized OFL system exhibited remarkable efficiency in treating pharmaceutical wastewater, with a chemical oxygen demand (COD) removal efficiency of 98.9% and an ammonia nitrogen removal efficiency of 90.8% under solar radiation.

## 1. Introduction

Solar-driven interfacial steam generation has emerged as a promising technology for diverse applications, including sterilization, desalination, and water purification [1]. The development of highly efficient photothermal materials is crucial to maximize the utilization of natural sunlight. To date, significant progress has been made in exploring various photothermal materials, including natural materials [2,3,4,5], carbon-based materials [6,7,8,9], metallic plasmonic materials [10,11,12], semiconductor materials [13,14,15,16], porous polymers [17,18,19,20,21], and composite materials [22,23,24]. Despite these advancements, the development of materials that simultaneously exhibit high photothermal conversion efficiency, cost effectiveness, and environmental friendliness remains a significant challenge [25,26].

Carbon materials derived from biomass carbonization have gained considerable attention due to their natural micro- and macro-structures, high hydrophilicity, and excellent light-harvesting capabilities. Various plant-derived materials, such as mushrooms [27], lotus seedpods [28], rice leaves [29], sunflower heads [30], Enteromorpha prolifera [31], daikon [32], loofah [33], sweet lime peels [34], kelp [35], tofu [26], carrot [36], and rice straw [37], have been successfully employed as photothermal materials after carbonization [38,39]. These carbonized plant materials not only exhibit broadband light absorption and efficient photothermal conversion but also retain the unique structural features of plants that facilitate water transport. Particularly, leaves have attracted significant interest for solar steam generation due to their exceptional water transport and light-harvesting capabilities [40]. For instance, carbonized lotus leaves have demonstrated remarkable SSG performance with an evaporation rate of 3.1 kg·m^−2^·h^−1^ [41].

Among various leaf structures, Osmanthus fragrans leaves (OFLs) are particularly noteworthy. As the primary structure for transpiration in evergreen broad-leaved vascular tree species [42], OFLs account for more than 90% of water evaporation [43]. The unique straight-hole structure within OFLs facilitates multiple light scattering and absorption while providing efficient pathways for moisture and vapor transmission, both of which are crucial for SSG processes [44]. The non-toxicity, stability, natural transpiration properties, excellent structural features, and hydrophilicity of OFLs make them an ideal source for photothermal materials. Consequently, carbonized OFL presents significant potential as a continuous and porous 3D network carbon sponge for solar-driven water transpiration applications.

The integration of different materials to form composites can yield synergistic effects, potentially enhancing photothermal efficiency beyond that of the individual components. For example, carbonized radish coated with titanium nitride and titanium oxide demonstrated significantly higher evaporation rates compared to pristine biomass carbon materials [29,45]. The combination of carbon materials with suitable semiconductors can create synergistic effects that not only improve photothermal conversion efficiency but also enable pollutant degradation through photocatalytic processes. Semiconductor materials like TiO_2_ are particularly promising due to their light absorption and hydrophilic properties under UV irradiation [46]. Recent studies have reported composite materials combining TiO_2_-nanoparticle-based photocatalytic functions with Au-nanoparticle-based plasmonic evaporation [47], as well as compound films composed of Au@TiO_2_ core-shell nanoparticles that enhance solar water evaporation [48]. The TiO_2_ in the composite can significantly increase the energy transfer efficiency under sunlight due to the light absorption and hydrophilic properties with UV irradiation [49]. A novel composite coagulation technique with built-in electric potential was proposed to fabricate high-performance uniaxial moisture-driven generators [50]. This study rationally designed a manganese oxide/poly-L-lysine co-modified carbon fiber cloth (CFC) composite that achieves high-efficiency antifouling solar-driven desalination through enthalpy reduction of water evaporation coupled with synergistic enhancement of broadband light absorption and antibacterial efficacy [51].

In this study, we developed a novel TiO_2_-decorated three-dimensional carbon material derived from carbonized Osmanthus fragrans leaves (TiO_2_/carbonized OFL) using biotemplating and hydrothermal methods. The TiO_2_/carbonized OFL composite was designed to synergistically combine the advantages of carbon and TiO_2_, while preserving the inherent porous structure of plant leaves. We systematically compared the solar evaporation efficiency of TiO_2_/carbonized OFL with carbonized OFL and OFL-templated TiO_2_ to demonstrate the enhancement in solar evaporation performance. Furthermore, we applied TiO_2_/carbonized OFL in the treatment of actual pharmaceutical wastewater under natural solar radiation, demonstrating its potential for practical water purification applications. This work not only establishes TiO_2_/carbonized OFL as a promising solar photothermal material but also provides new insights for future research in solar-driven wastewater treatment technologies, particularly through the synergistic combination of TiO_2_ and carbonized biomass.

## 2. Experimental

### 2.1. Materials

All chemicals were analytical grade and could be used without further purification. The osmanthus leaves (OFLs) used in this study were collected from pruning waste on the campus of Yunnan University during autumn. These selected mature leaves exhibited a healthy green coloration without parasitic infestation, with an average dimension of 4 cm × 11 cm. Anhydrous glucose (C_6_H_12_O_6_, AR) was purchased from Tianjin Fengchuan Chemical Reagent Technology Co., Ltd. (Tianjin, China); isopropyl alcohol titanium (Ti_4_(OCH_3_)_16_, 98%) was purchased from Beijing Inocai Technology Co., Ltd. (Beijing, China); glutaraldehyde (C_5_H_8_O_2_, 50%) was purchased from Tianjin Damao Chemical Reagent Factory; and acetylacetone (C_5_H_8_O_2_, 99%) was purchased from Guangdong Guanghua Technology Co., Ltd. (Guangzhou, China). Pharmaceutical wastewater was collected from a production facility in Songming, Yunnan Province.

### 2.2. Synthesis of Carbonized OFL

The Osmanthus leaves were cut into small 1 cm × 1 cm pieces and dried. Then, a certain mass of dried osmanthus leaves (OFLs) was mixed with 60 mL of deionized water, and 3% mass fraction of glucose was added. The glucose was stirred until it was completely dissolved, and the mixture was heated in a water bath autoclave reactor at 200 °C for 24 h. After that, the prepared material was dialyzed in pure water for 2–3 days, followed by freeze-drying for 48 h to obtain carbonized OFL material.

### 2.3. Synthesis of TiO_2_/OFL Composites

The preparation method of TiO_2_/OFL composite materials combines biological templates with hydrothermal synthesis, using OFL as a biological template carrier and TiO_2_ as the precursor solution. The osmanthus leaves (OFLs) were cut into appropriate sizes and then soaked in a 4% glutaraldehyde solution for 24 h to fix the plant template. Then, it was rinsed with pure water 3 to 4 times and placed at room temperature to remove surface moisture. Then, gradient dehydration of the material was performed using 30%, 50%, 70%, 90%, and 100% ethanol solutions. The dehydrated material was soaked in 10% HCl for 24 h, then washed with pure water until neutral, and finally soaked in ethanol for 2 h. After that, the materials used to remove the surface ethanol solution were placed in a fume hood to air dry naturally at room temperature for about one week, resulting in the OFL biological template carrier. 

To obtain the Ti precursor solution, 10 mL of 98 wt% TTIP solution, 190 mL of 99 wt% acetylacetone, and 1 mL of anhydrous ethanol were mixed. The ratio of TTIP to acetylacetone was chosen to ensure optimal TiO_2_ nanoparticle formation during the hydrothermal process. An appropriate amount of air-dried OFL biotemplate carrier was weighed and immersed in the precursor solution for 24 h. The TiO_2_/OFL composite material was freeze-dried to form it. After that, the material soaked in the precursor solution was placed in a quartz boat and heated in a tube furnace under an N_2_ atmosphere, with a programmed heating rate of 5 °C/min until reaching 280 °C, where it was held for 2 h. Then, it continued to warm to 460 °C for 4 h to obtain the TiO_2_/carbonized OFL composite materials.

### 2.4. Solar Steam Generation

For the solar evaporation experiment, a solar simulator was used to test three different types of materials, namely OFL, carbonized OFL, and TiO_2_/OFL composites. These materials were cut into disc shapes and immersed in a container filled with water for a solar evaporation experiment. To prevent heat loss, a layer of cotton fiber was wrapped around the outer wall of the container (glass bottle). The solar evaporation rate was measured under different solar radiation intensities (1 kW/m^2^, 3 kW/m^2^, 5 kW/m^2^, 10 kW/m^2^). An analytical electronic balance was used to record the change in water mass (Δm) during the evaporation process in sunlight at 15 min intervals, while monitoring the temperature (T) of the material with an infrared imaging thermometer. The evaporation rate (η) is calculated as follows:

*η* = Δ*m*/(*T* × *S*)

In this, η represents the evaporation rate, measured in kg·m^−2^·h^−1^; Δm is the change in water weight, measured in kg; T is time, measured in hours; and S is the cross-sectional area of the corresponding material, measured in m^2^. Please note that the diameter of each material is 0.01 m, so S remains constant in this study.

### 2.5. Characterization

The phase composition of the samples was characterized using a TTR-III X-ray diffractometer (XRD) (Rigaku Corporation, Tokyo, Japan) with Cu Kα radiation (λ = 0.15406 nm), performing continuous scanning in the 2θ range of 10–90°. Elemental composition and chemical states were investigated by X-ray photoelectron spectroscopy (XPS) on a K-Alpha+ system (Thermo Fisher Scientific Inc., Waltham, MA, USA), where the binding energy scale was calibrated using the adventitious carbon C 1s peak at 284.8 eV, followed by peak deconvolution analysis with Avantage 5.9 software. Morphological features were examined using an FEI Nova NanoSEM450 field-emission (FEI Company, Hillsboro, OR, USA) scanning electron microscope (SEM) operated at 15 kV. Optical absorption properties were evaluated by UV-vis-NIR spectroscopy (Shimadzu Corporation UV-2401PC, Kyoto, Japan) over the spectral range of 200–2500 nm. Surface functional groups were identified through Fourier-transform infrared spectroscopy (FTIR) measurements conducted on a Thermo Nicolet 8700 spectrometer (Thermo Fisher Scientific Inc., Waltham, MA, USA) equipped with a diamond ATR accessory, collecting 32 scans at a 4 cm^−1^ resolution.

## 3. Results and Discussion

### 3.1. Synthesis and Characterization

**Figure 1:**
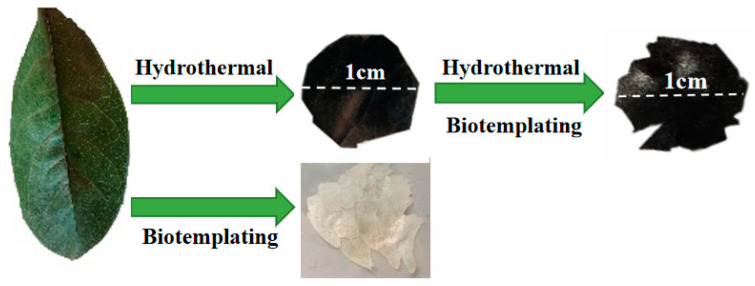
Intuitive images of natural OFL, carbonized OFL, TiO_2_/OFL, and OFL-templated TiO_2_.

Pre-treatment of biomass before pyrolysis is considered an effective way to improve the properties of biochar [52]. Vacuum freeze-drying has been proven to be an effective pre-treatment method [53]. During the freeze-drying process, the transformation through water-ice-sublimation improved the crystalline structure and hydrophobicity of the biomass, resulting in biochar with a uniform pore surface structure after pyrolysis [54]. Hydrothermal pretreatment is also a type of pretreatment method. Hydrothermal pretreatment can enhance the specific surface area, pore volume, content of oxygen-containing functional groups, and crystallinity of biochar. In our work, a facile hydrothermal method was developed to prepare carbonized OFL. The primitive pore structure of the leaves was preserved by controlling the temperature and pyrolysis time while carbonizing. At the same time, glucose solution as a hydrothermal solvent can establish chemical engineering of the material surface (such as improving the hydrophilicity of the OFL). The combination of biotemplating and hydrothermal methods enables the uniform growth of TiO_2_ on both the inner and outer surfaces of the material. On the basis, we introduced the biotemplating method in the preparation process of carbonized OFL to obtain a TiO_2_/OFL composite that could fully replicate the surface and internal structure of OFL. The intuitive images of carbonized OFL, TiO_2_/OFL, OFL temp-TiO_2_, and their precursors can be seen in Figure 1. The carbonized OFL and TiO_2_/OFL were black in color, and the OFL-templated TiO_2_ were white; they all inherited the flake shape of the natural leaf.

**Figure 2:**
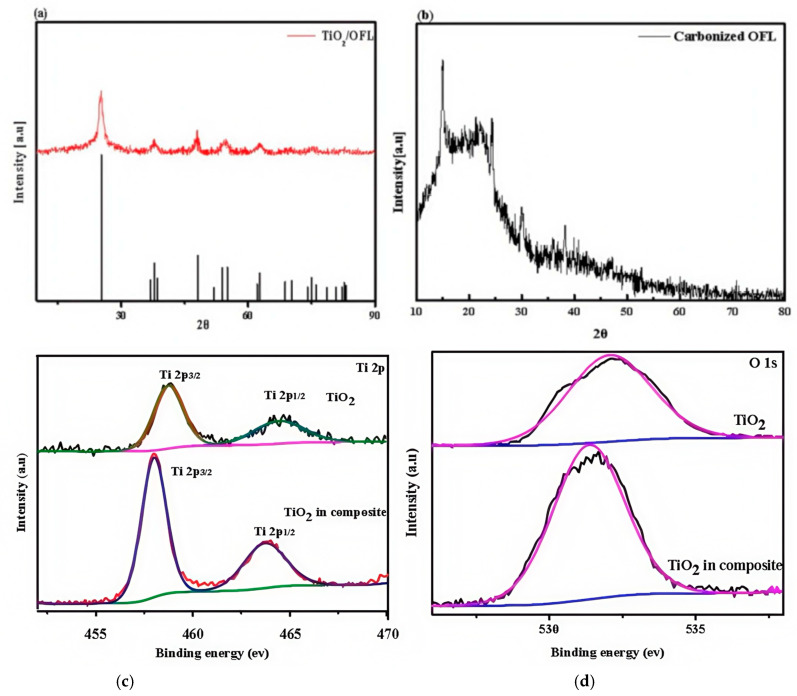
(**a**) XRD pattern of the TiO_2_/OFL; (**b**) XRD pattern of the carbonized OFL; (**c**,**d**) XPS spectra of TiO_2_/OFL.

The XRD pattern of the TiO_2_/OFL composite is presented in Figure 2a, showing distinct diffraction peaks at 2θ ≈ 25°. Distinct diffraction peaks are observed, with the most prominent peak appearing at 2θ ≈ 25°, corresponding to the (101) crystal plane of anatase TiO_2_ (JCPDS No. 21-1272). The well-defined position and intensity of this characteristic peak confirm the crystalline nature of TiO_2_ in the composite material. Furthermore, additional diffraction peaks corresponding to other crystal planes of anatase TiO_2_, including (004), (200), (105), and (211), are clearly identified, providing strong evidence for the successful incorporation of crystalline TiO_2_ onto the OFL biochar matrix. In the higher 2θ range (2θ > 60°), the diffraction pattern shows a gradual decrease in peak intensity with the emergence of a broad background, suggesting the coexistence of amorphous carbon phases within the composite structure.

For comparison, Figure 2b displays the XRD pattern of pristine OFL biochar, which exhibits a broad diffraction hump centered around 2θ ≈ 24°, characteristic of amorphous carbon materials. This pattern indicates that the high-temperature carbonization of osmanthus leaves primarily yields biochar with a disordered carbon structure, lacking long-range crystalline order. The distinct contrast between the XRD patterns of TiO_2_/OFL composite and pure OFL biochar clearly demonstrates the successful integration of crystalline TiO_2_ nanoparticles with the amorphous carbon matrix through the composite preparation process.

The XPS analysis of the TiO_2_/OFL composite provides detailed insights into its chemical composition and bonding states. Figure 2c presents the high-resolution Ti 2p spectrum, which exhibits two well-defined peaks at binding energies of 458.5 eV and 464.3 eV, corresponding to Ti 2p^3/2^ and Ti 2p^1/2^, respectively. These peaks are characteristic of the Ti^4+^ oxidation state, confirming the presence of TiO_2_ in the anatase phase. The spin-orbit splitting energy of 5.8 eV between the Ti 2p^3/2^ and Ti 2p^1/2^ peaks is consistent with the reported values for crystalline TiO_2_, further validating the successful incorporation of TiO_2_ into the composite material.

Figure 2d displays the deconvoluted O 1s spectrum, which reveals the complex oxygen environment within the composite. The spectrum can be resolved into three distinct components: (1) a dominant peak at 530.0 eV, attributed to lattice oxygen in the Ti-O bonds of TiO_2_; (2) a secondary peak at 531.5 eV, where the oxygen associated with C=O and -OH functional groups may originate from the biochar matrix; and (3) a minor peak at 533.0 eV, corresponding to weakly bonded oxygen species such as C-O or adsorbed water molecules. The presence of these oxygen species suggests a synergistic interaction between the TiO_2_ nanoparticles and the oxygen-rich functional groups on the biochar surface. This multifunctional oxygen environment not only enhances the material’s surface reactivity but also potentially improves its performance in applications such as photocatalytic water treatment or solar-driven evaporation, where surface chemistry plays a critical role. The coexistence of Ti-O bonds and oxygen-containing functional groups further confirms the successful integration of TiO_2_ with the biochar matrix, creating a composite material with tailored surface properties.

**Figure 3:**
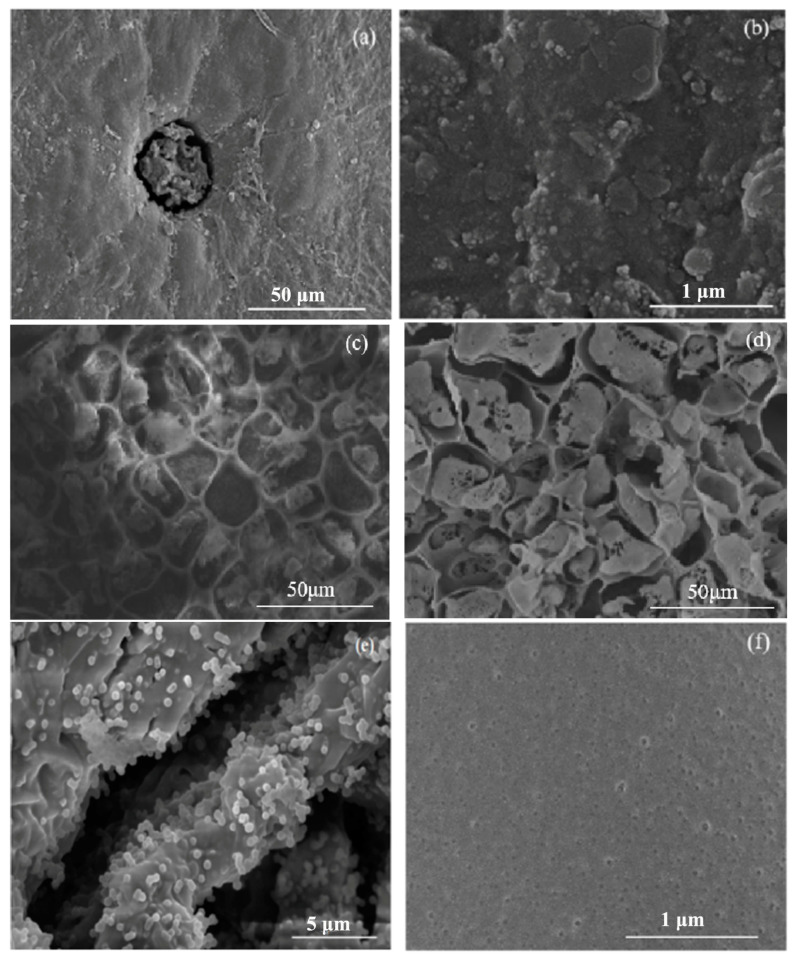
(**a**,**b**) The SEM surface morphology of natural OFL; (**b**) the morphology of natural OFL at 100,000 times magnification; (**c**,**d**) SEM surface of carbonized OFL morphology diagram; and (**e**,**f**) SEM surface morphology of TiO_2_/carbonized OFL, where (**f**) is the SEM surface morphology of TiO_2_/carbonized OFL at 100,000 times magnification.

The morphological evolution of the materials at different stages of processing is presented in Figure 3. Figure 3a reveals the surface structure of natural osmanthus fragrans leaves (OFL), exhibiting a characteristic rough fibrous surface with naturally occurring pores, which are intrinsic to the plant’s native morphology. A higher magnification view in Figure 3b provides further insight into the microstructure, revealing the presence of surface particulate matter. The transformation upon carbonization is evident in Figure 3c,d, where the carbonized OFL displays a well-defined, hierarchical porous structure with uniform pore distribution and enhanced surface openness, compared to its natural counterpart. The TiO_2_-modified OFL, as shown in Figure 3e,f, demonstrates successful surface functionalization, with TiO_2_ nanoparticles uniformly dispersed across the substrate. The high-resolution image (100,000×) in Figure 3f reveals a dense and homogeneous distribution of TiO_2_ particles, suggesting effective surface modification. This nanostructured surface is particularly advantageous for photocatalytic applications and may significantly influence the material’s wettability properties.

Due to the agglomerated nanoparticles of pure TiO_2_ [55], the introduction of OFL in the TiO_2_/carbonized OFL composite significantly suppresses particle aggregation, resulting in more dispersed and smaller particles. This reduction in particle size and improved dispersion can enhance the solar-driven water evaporation rate, expose more active sites, and promote pollutant adsorption and degradation.

Notably, the modification process has preserved and enhanced the natural stomatal structures of the leaf, as evidenced by the high-magnification images. The removal of the natural waxy coating during processing has exposed these stomatal features, which are known to enhance light-trapping capabilities [56,57,58,59]. The replication of these biological structures in the TiO_2_/carbonized OFL composite is particularly advantageous for solar-driven applications, as it combines the light-harvesting efficiency of natural plant structures with the photocatalytic properties of TiO_2_.

**Figure 4:**
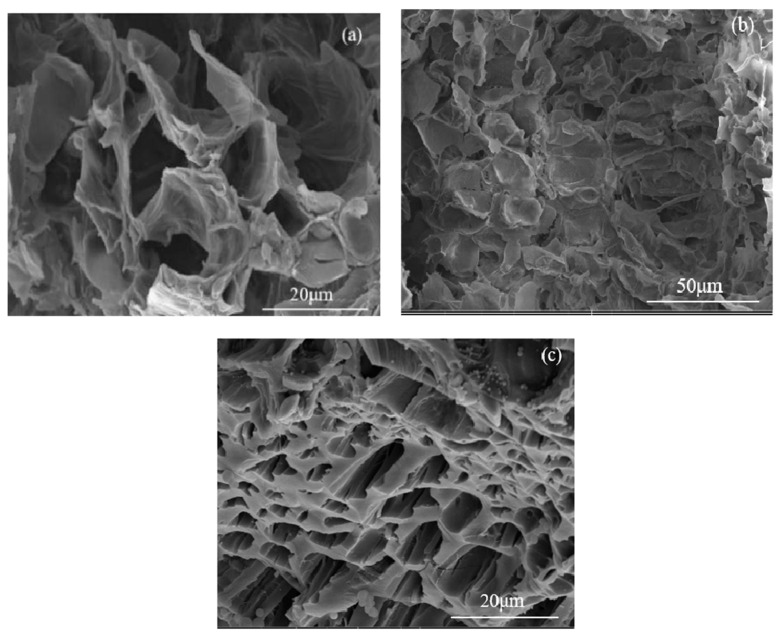
(**a**) The internal micro SEM morphology of natural OFL; (**b**) the internal micro SEM morphology of carbonized OFL; and (**c**) the internal micro SEM morphology of TiO_2_/carbonized OFL.

Cross-sectional analysis (Figure 4) further confirms that both carbonized OFL and TiO_2_/carbonized OFL maintain the intrinsic straight-pore architecture of natural OFL, while demonstrating improved structural regularity and channel alignment. This preservation of the natural vascular structure, combined with the introduced modifications, creates an optimized system for water transport and light absorption. The biomimetic approach employed in this study successfully translates the evolutionary advantages of natural leaf structures into engineered materials, providing an effective structural foundation for enhanced solar steam generation (SSG) performance. The hierarchical porosity, from macro-scale channels to nano-scale features, facilitates efficient water transport while maximizing light absorption and vapor generation capabilities.

### 3.2. Evaporation Performance of the Materials

To evaluate the photothermal performance, the materials were subjected to simulated solar irradiation at varying power densities (1 sun, 3 suns, 5 suns, and 10 suns) in a controlled experimental setup. The mass changes of water were monitored at 10 min intervals over a 1 h period. For comparative analysis, pristine OFL, carbonized OFL, TiO_2_/carbonized OFL, and pure water were tested under identical environmental conditions, maintained at an ambient temperature of 24 °C and relative humidity of 45%.

**Figure 5:**
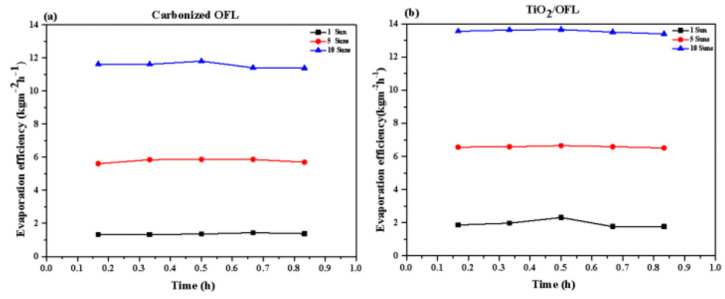
(**a**) Changes of the evaporation rate of the carbonized OFL under different light intensities with time; and (**b**) changes of the evaporation rate of the TiO_2_/carbonized OFL under different light intensities with time.

The experimental results, as summarized in the accompanying table and illustrated in Figure 5, demonstrate the water evaporation rates for pure water, carbonized OFL, and TiO_2_/carbonized OFL composite under different solar intensities (1 sun, 5 suns, and 10 suns). The data reveal that both TiO_2_/carbonized OFL and carbonized OFL exhibit significantly enhanced evaporation rates compared to natural OFL and pure water across all tested light intensities.

**Figure 6:**
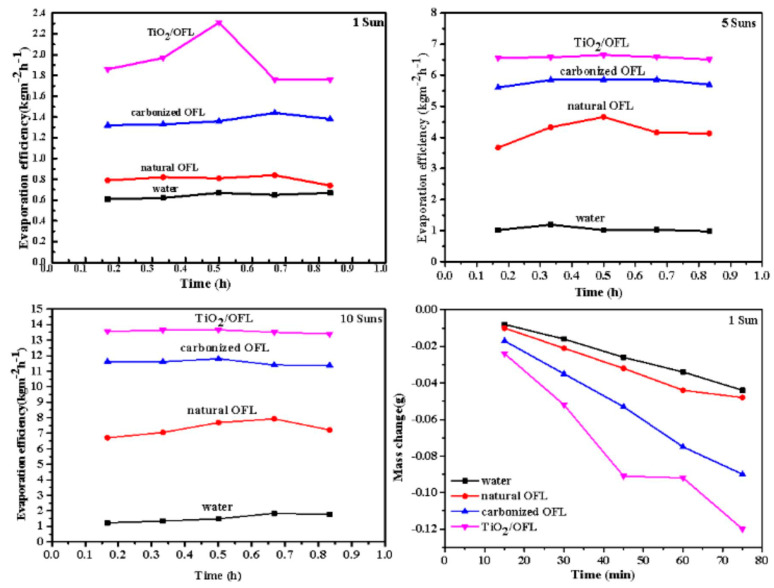
Transpiration efficiency of carbonized OFL and TiO_2_/carbonized OFL under different light intensities as a function of evaporation time.

As shown in Figure 6, the variation of transpiration efficiency with evaporation time for TiO_2_/TiO_2_ OFL and TiO_2_/TiO_2_ OFL under different light intensities is presented. Under 1-sun irradiation (1 kW/m^2^), the TiO_2_/carbonized OFL composite demonstrated remarkable performance, with a cumulative mass loss of 0.12 g after 50 min of illumination, significantly outperforming previously reported carbonized biomass materials (e.g., carbonized lotus leaves with an evaporation rate of 3.1 kg·m^−2^·h^−1^). This represents a 3-fold and 1.3-fold increase in evaporation rate compared to pure water and carbonized OFL, respectively. The superior performance of TiO_2_/OFL can be attributed to the synergistic combination of the biological template, TiO_2_ photocatalyst, and carbon matrix, which collectively enable efficient water transport and enhanced light absorption. The black surface of the composite further contributes to its excellent photothermal conversion efficiency. These results clearly demonstrate that the TiO_2_/carbonized OFL composite, with its optimized structural and compositional design, significantly outperforms simply carbonized materials in solar steam generation applications.

**Figure 7:**
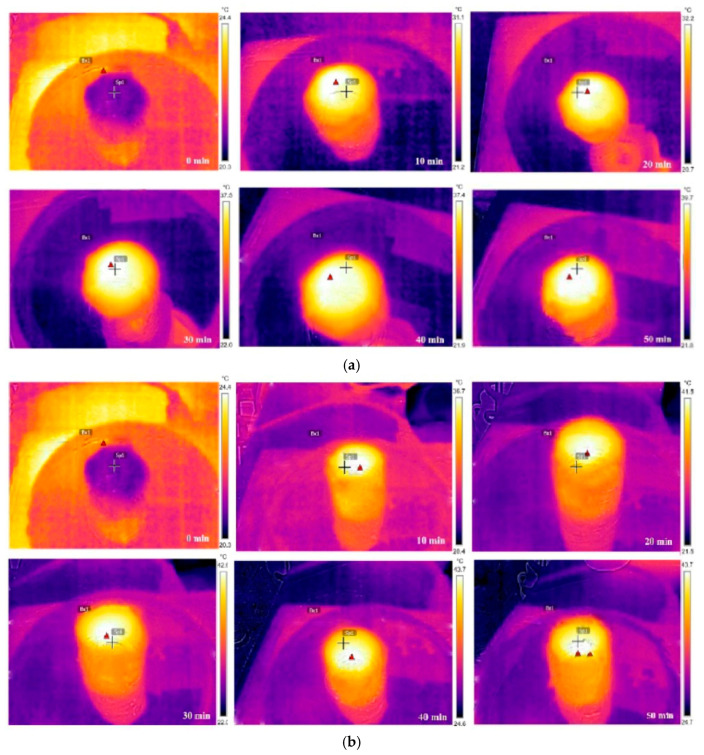
(**a**) Infrared images of carbonized OFL under an irradiation intensity of 1 sun. (**b**) Infrared images of TiO_2_/carbonized OFL under an irradiation intensity of 1 sun.

Temperature changes were monitored using infrared (IR) thermal imaging. Two sets of infrared images illustrate the temperature variations of the carbonized OFL and TiO_2_/carbonized OFL under 1-sun illumination during the experiment (Figure 7a,b). The images were taken from a top view. As shown in Figure 7b, the surface temperature of TiO_2_/carbonized OFL reached 41.5 °C from room temperature within approximately 20 min, and the temperature steadily increased and reached 43.7 °C after 50 min. However, the surface temperature of the carbonized OFL only increased to 39.7 °C after being exposed to sunlight for 50 min. The surface temperature of TiO_2_/carbonized OFL changed more rapidly than that of carbonized OFL and reached the highest value under the same irradiation time. Additionally, as TiO_2_ particles grow on both the surface and interior of the composite, thin water films form on these areas. This setup offers two key advantages: (i) The TiO_2_ particles and carbon on the top layer absorb solar energy multiple times, heating the water film on the surface and causing a sharp rise in temperature. (ii) Due to the linear microchannels and pinholes in the OFL, convection occurs between the hot water in the top layer and the colder water at the backside. This facilitates heat transfer through the channels until thermal equilibrium is reached. This indicates that TiO_2_/carbonized OFL can effectively absorb and convert solar energy into heat with less heat loss to the bulk water.

To evaluate the performance of different solar-driven water evaporation materials, we compared three materials, analyzing their synthesis methods, water evaporation rates, advantages, and limitations (Table 1). The results indicate that all these materials exhibit high photothermal conversion efficiency but differ in stability, fabrication complexity, and practical applicability.

Among them, PVA@PCLS, synthesized using the sol-gel method, achieved the highest water evaporation rate (2.33 kg·m^−2^·h^−1^) [60]. This excellent performance can be attributed to its efficient solar-to-vapor conversion capability and well-structured porous architecture. Additionally, the utilization of biomass-derived precursors enhances resource recycling. However, the preparation of biochar via the sol-gel method requires high energy input, which may limit its large-scale application. Furthermore, its long-term stability and durability in practical environments remain to be evaluated.

The CFC/MnO_2_/PLL composite, prepared via electrostatic adsorption, exhibited a slightly lower evaporation rate (2.20 kg·m^−2^·h^−1^) [51]. The primary advantages of this material include its reduced water evaporation enthalpy, which facilitates efficient energy utilization, and its enhanced light absorption properties, which contribute to a higher evaporation rate. Additionally, the incorporation of MnO_2_ endows the material with antibacterial properties, making it more promising for water treatment applications. However, its long-term durability remains uncertain, and the synthesis process involves multiple steps, making it more complex and costly compared to other methods.

The TiO_2_/carbonized OFL composite, synthesized through the hydrothermal method, exhibited an evaporation rate of 2.31 kg·m^−2^·h^−1^, which is comparable to that of PVA@PCLS. This material demonstrates high stability and excellent water purification capability, making it a promising candidate for integrated water treatment applications. The hydrothermal synthesis method allows for well-dispersed TiO_2_ nanoparticles on the carbonized substrate, enhancing both photothermal and photocatalytic properties. However, its applicability to diverse pollutants is still limited, which may constrain its effectiveness in treating complex wastewater compositions.

From the above comparison, it is evident that each material has distinct strengths and weaknesses. While PVA@PCLS shows the highest evaporation rate, its high energy consumption during fabrication may hinder large-scale deployment. CFC/MnO_2_/PLL introduces antibacterial properties and energy-efficient evaporation, yet its high synthesis cost and potential durability issues require further optimization. TiO_2_/carbonized OFL offers a balance between high stability and water purification ability but needs further modifications to broaden its pollutant removal capacity. Future research should focus on optimizing synthesis methods to reduce fabrication energy consumption and cost while maintaining high evaporation efficiency. Additionally, improving the long-term stability and broadening the water purification capabilities of these materials are essential for practical solar-driven water treatment applications.

**Figure 8:**
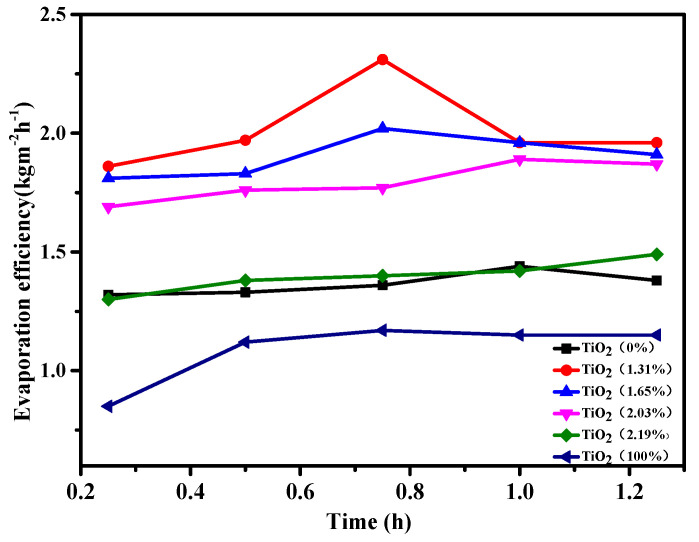
The evaporation performance of the TiO_2_/carbonized OFL composite with different amounts of TiO_2_.

To quantitatively study the solar-driven evaporation performance of the TiO_2_/carbonized OFL composite with different content of the TiO_2_, we changed the addition ratio of the titanium source in the precursor solution to prepare six groups of composite materials with different TiO_2_ content. The water evaporation performance test and XPS characterization of the material were carried out. The results are shown in Figure 8. The composite material with TiO_2_ content of 1.31% exhibited the highest water evaporation efficiency, and the maximum water evaporation rate can reach 2.31 kg·m^−2^·h^−1^. The composite materials with TiO_2_ content of 1.65% and 2.03% also have good water evaporation performance, and the water evaporation rate can reach 2.02 kg·m^−2^·h^−1^ and 1.89 kg·m^−2^·h^−1^, respectively. With the increase of the proportion of titanium source in the precursor solution, when the TiO_2_ content of the composite material is 2.19%, the water evaporation rate decreases significantly, making it even lower than that of carbon materials. As a comparison, the evaporation efficiency of the OFL-templated pure TiO_2_ without carbon source was also tested in the same condition. The evaporation efficiency of SSG with OFL-templated pure TiO_2_ is very low and close to the efficiency of pure water. The photothermal conversion property of the composite material, consisting of biochar and titanium dioxide, initially increases with the loading but then decreases as the load continues to rise. This is because titanium dioxide particles with excessively large diameters hinder the interaction between water molecules and the heated components. Additionally, these larger particles may obstruct the movement of water molecules. Therefore, we can conclude that the water evaporation performance of TiO_2_/carbonized OFL depends on two key factors: the structure of the material and the relative content of TiO_2_ in the composite.

### 3.3. UV-Vis-NIR Diffuse Reflectance Spectra

**Figure 9:**
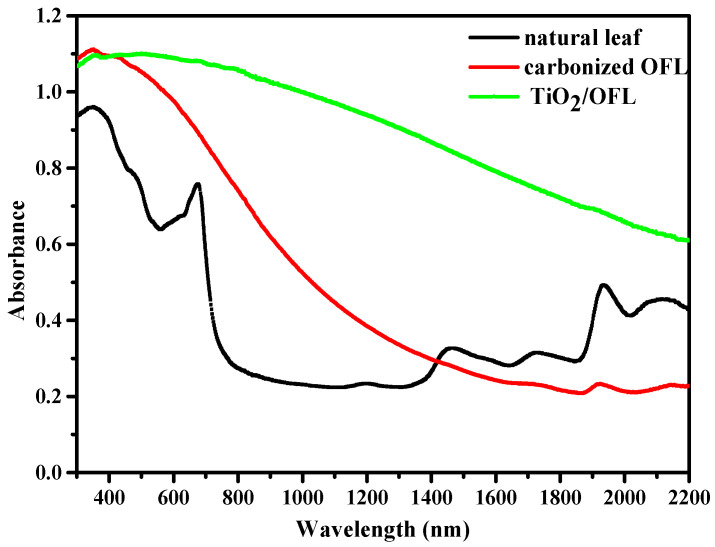
The UV-Vis diffuse reflectance spectra of carbonized OFL and TiO_2_/carbonized OFL.

Light absorption properties of the materials were measured in the solar spectrum range (wavelengths from 300 to 2200 nm). A comparison of the absorption spectra of carbonized OFL and TiO_2_/carbonized OFL is presented in Figure 9. The carbonized OFL exhibits higher absorbance in the ultraviolet region, with relatively stable absorbance around 400–600 nm, close to 1. As the wavelength increases, the absorbance gradually decreases, particularly beyond 800 nm, where it significantly declines and remains low after 1200 nm. In contrast, TiO_2_/carbonized OFL shows minimal changes in absorbance across the entire wavelength range, with a gentle downward trend. Its absorbance remains relatively high in the 400–1200 nm range, demonstrating excellent broadband absorption characteristics. While carbonized OFL has strong absorbance in the ultraviolet region, its absorbance rapidly diminishes in the near-infrared region. On the other hand, TiO_2_/carbonized OFL maintains relatively high absorbance over a broader wavelength range, exhibiting superior broadband absorption in both the ultraviolet and visible light regions, which is more favorable for applications such as photocatalysis. In summary, compared to carbonized OFL, TiO_2_/carbonized OFL demonstrates better absorption over a wider spectral range, while carbonized OFL excels in the ultraviolet region but performs poorly in the near-infrared region. First of all, the structures of micron/submicron holes as well as the rough surface of materials enable strong and broad optical resonances, resulting in efficient light trapping and absorption enhancement of carbon-based materials [44]. At the same time, due to the continuous scattering and refraction of light incident on the composite film containing TiO_2_ crystals in the Au@TiO_2_ core-shell nanoparticle structure, the light absorption performance of carbon is enhanced [48]. Based on the abovementioned effects, the solar evaporation improved with the use of the TiO_2_/carbonized OFL composites.

### 3.4. FT-IR Spectra

**Figure 10:**
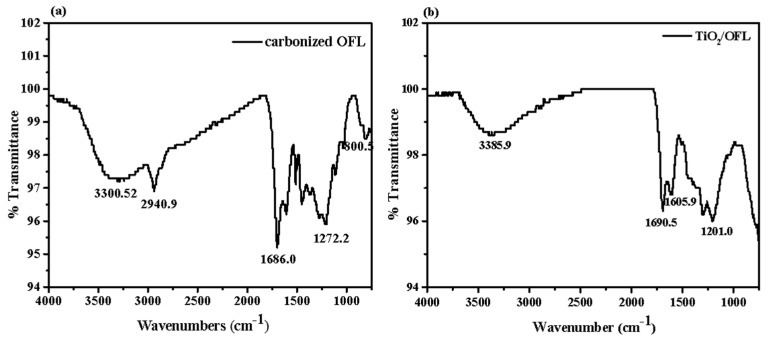
Fourier infrared spectrum of the material in the range of 500–4000 nm; (**a**) shows the Fourier infrared absorption spectrum of the carbonized OFL; (**b**) shows the Fourier infrared absorption spectrum of the TiO_2_/carbonized OFL.

The Fourier-transform infrared (FTIR) spectra in Figure 10 illustrate the transmittance variations of carbonized OFL and TiO_2_/carbonized OFL in the wavenumber range of 500–4000 cm^−1^. Figure 10a represents the FTIR spectrum of carbonized OFL. The band at 3300.52 cm^−1^ corresponds to O-H stretching vibrations, attributed to hydroxyl groups or residual water on the material surface. The peak at 2940.9 cm^−1^ is characteristic of C-H stretching vibrations, indicating the presence of hydrocarbon groups in the material. The band at 1686.0 cm^−1^ is typically associated with C=O stretching vibrations, suggesting the presence of carbonyl groups. The bands at 1272.2 cm^−1^ and 800.4 cm^−1^ are possibly linked to vibrations of C-O or other C-X bonds, which might originate from oxygen-containing functional groups formed during the carbonization process. Figure 10b shows the FTIR spectrum of TiO_2_/carbonized OFL. The peak at 3385.9 cm^−1^ falls within the typical range of O-H stretching vibrations, originating from hydroxyl groups or water on the TiO_2_ surface. The band at 1690.5 cm^−1^ corresponds to the bending vibrations of C=O or O-H bonds. The band at 1201.0 cm^−1^ is likely associated with the stretching vibrations of C-O or Si-O bonds, potentially arising from functional groups in organic compounds or inorganic oxides.

A comparative analysis reveals that both samples exhibit O-H stretching vibration peaks in the range of 3300–3385 cm^−1^, with TiO_2_/carbonized OFL showing a higher peak, potentially indicating a higher surface hydration or hydroxyl content. For C=O and C-H absorptions, carbonized OFL exhibits more pronounced peaks for C=O (1686.0 cm^−1^) and C-H (2940.9 cm^−1^), which are characteristic of hydrocarbons and carbonyl groups formed during carbonization. These peaks are weaker or absent in TiO_2_/carbonized OFL. In the low-wavenumber region (1200–800 cm^−1^), both carbonized OFL and TiO_2_/carbonized OFL display distinct characteristic peaks, likely originating from stretching vibrations of C-O, Si-O, or Ti-O bonds. These peaks reflect the compositional and structural differences between the two materials. In summary, carbonized OFL exhibits more prominent carbon-based functional group characteristics, while TiO_2_/carbonized OFL demonstrates more surface features related to TiO_2_.

### 3.5. Purification of Pharmaceutical Wastewater

**Figure 11:**
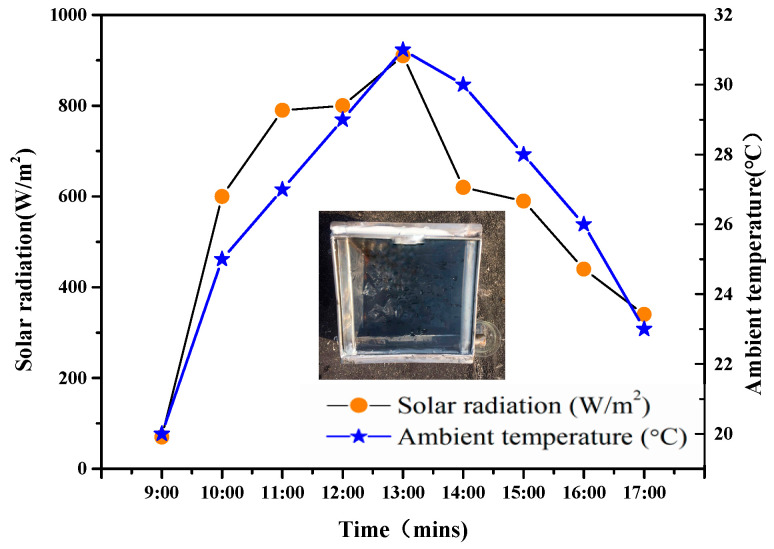
Variations in solar irradiance and ambient temperature over an 8 h period in a practical solar-driven evaporation wastewater treatment system.

The carbonized OFL and TiO_2_/carbonized OFL composite were applied for evaporative purification of pharmaceutical wastewater under 8 h solar irradiation. As shown in Figure 11, we fabricated a device designed to directly generate clean water using solar energy. This device consists of two compartments: one for contaminated water and another for condensed purified water. The side walls were constructed with steel plates equipped with thermal insulation layers to minimize solar absorption losses. The top of the device was covered with glass. The carbonized OFL and TiO_2_/carbonized OFL composite floated at the air–water interface of the contaminated solution to absorb solar energy.

During the experiment, solar steam generation was influenced by climatic parameters including solar irradiance, ambient temperature, and wind speed. Solar irradiance was recorded throughout the process using a pyranometer. The temperature of the top glass surface exceeded the ambient temperature. The ambient temperature affected the temperature difference (ΔT) between the bulk water in the basin and the inner surface of the glass cover. Wind speed enhanced the natural condensation process in the solar steam generation system by reducing the ambient temperature around the system and slightly cooling the top glass surface for condensation. The average solar irradiance and ambient temperature were 573 W/m^2^ and 26.6 °C, respectively.

**Figure 12:**
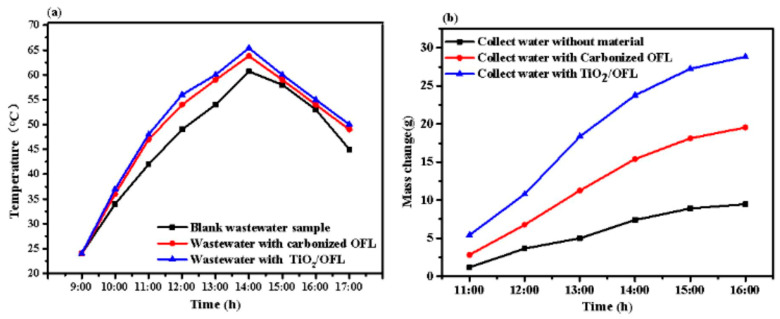
(**a**) The change of wastewater surface temperature in evaporation experiment; (**b**) the amount of purified water collected in the 8 h evaporation experiment.

Figure 12a shows the surface water temperature in the solar steam generation (SSG) system under real solar irradiance, recorded by thermocouples. During the 8 h pharmaceutical wastewater evaporation treatment, the water temperature in the TiO_2_/carbonized OFL device remained consistently higher than that of the carbonized OFL and blank control groups. The peak surface water temperature reached 66 °C at 14:00 (with an irradiance of 0.62 kW/m^2^). The mass of collected purified water was measured using an electronic balance, as illustrated in Figure 12b. After 8 h of irradiation, approximately 30 mL of purified water was collected from the TiO_2_/carbonized OFL device, compared to 19 mL from the carbonized OFL device and only 9 mL from the blank group. The solar still equipped with TiO_2_/carbonized OFL achieved a productivity of 6.9 L/m^2^.

In solar-driven water evaporation research, selecting an appropriate experimental duration is crucial for evaluating system performance. Generally, the experimental duration should align with the daylight hours in actual applications to ensure the reliability and practical relevance of the results. The effective daylight hours typically range from 6 to 10 h, making an 8 h experimental duration a widely accepted choice. For example, in one study, researchers developed an environmentally friendly photothermal hydrogel evaporator for efficient solar-driven water purification and conducted a continuous 12 h test [61]. The results showed that the evaporation performance remained stable over the long duration, demonstrating the high reliability of the system. Additionally, previous studies have shown that TiO_2_ possesses excellent thermal stability. For instance, a study investigated the stability of electron-beam-deposited TiO_2_ monolayers and TiO_2_/SiO_2_ high-reflective layers during annealing at temperatures ranging from 300 to 1100 °C and found that the main optical properties of the material did not significantly degrade below 900 °C [62]. Furthermore, TiO_2_, as a widely used photocatalytic material, has been thoroughly validated for its heat resistance and chemical stability. Research has shown that TiO_2_ nanotube array films retain their tubular structure after being calcined at 650 °C for 2 h, further proving their excellent stability under high-temperature conditions [63]. In conclusion, an 8 h experimental duration effectively simulates system performance under actual sunlight conditions. Combining performance trend analysis, the inherent stability of the materials, and support from the relevant literature, it can be inferred that the TiO_2_/carbonized OFL system demonstrates good stability in long-term use.

**Figure 13:**
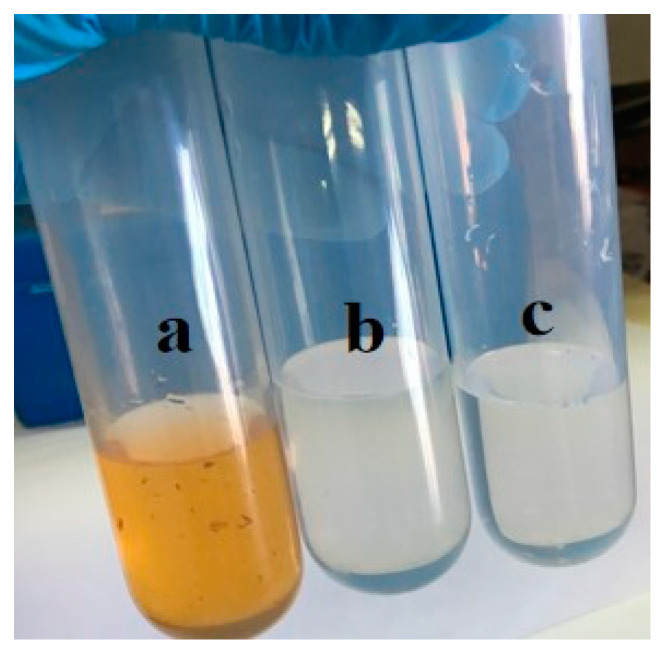
(**a**) Pharmaceutical wastewater; (**b**) pharmaceutical wastewater treated by carbonized OFL; and (**c**) pharmaceutical wastewater treated by TiO_2_/carbonized OF.

**Figure 14:**
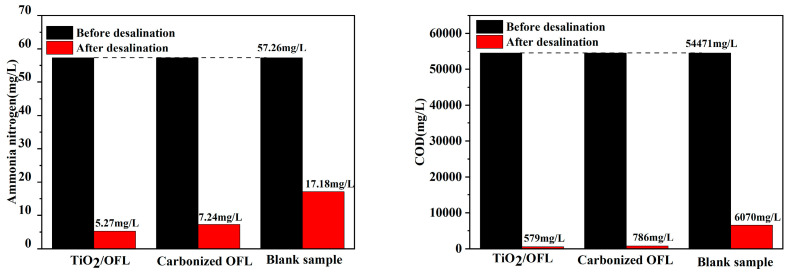
Changes in ammonia nitrogen and COD content before and after treatment of pharmaceutical wastewater with the materials prepared in this work.

As shown in Figure 13a, after purification by SSG, the color of the collected water is close to clear, indicating SSG based on TiO_2_/carbonized OFL and carbonized OFL can be effectively applied for removal of pollutants. Figure 14 shows the COD and ammonia nitrogen removal performance by carbonized OFL and TiO_2_/carbonized OFL composite. The SSG with TiO_2_/carbonized OFL was able to reduce COD concentrate and ammonia nitrogen concentrate from 54471 mg/L and 57.26 mg/L to 579 mg/L and 5.27 mg/L, respectively. The COD concentrate and ammonia nitrogen concentrate were also reduced to 786 mg/L and 7.24 mg/L for the carbonized OFL, while the collected purified water was 6070 mg/L (COD) and 17.18 mg/L (ammonia nitrogen) for the blank group. The SSG with carbonized OFL removed 98.5% of COD and 87.4% of ammonia nitrogen in the pharmaceutical wastewater after evaporation, indicating its good water purification capacity. By comparison, more than 98.9% of the COD and 90.8% of the ammonia nitrogen was removed by the SSG with TiO_2_/carbonized OFL. The enhanced performance should be attributed to the combined synergetic water purification effects. It is proved that the TiO_2_/carbonized OFL composite combining the advantage of TiO_2_ and carbon can not only improve the water evaporation performance of the SSG but also play a role in the further purification of water during the evaporation process.

## 4. Conclusions

In summary, a composite material incorporating TiO_2_ and porous carbon derived from OFL was designed for efficient clean water generation powered by abundant solar energy. The uniformly distributed TiO_2_ particles within the composite improve solar light absorption and facilitate the conversion of solar energy into localized heat at the interface. This heat directly drives the evaporation-condensation process, effectively distilling clean water from the contaminated solution. Compared with the carbonized OFL, the TiO_2_/carbonized OFL composite played a better role in promoting solar evaporation of water. The TiO_2_/carbonized OFL solar steam generator was able to generate steam at a rate of 2.31 kg·m^−2^·h^−1^ under 1-sun illumination, which is 3.45 times the bare water system. Moreover, the productivity of clean water of the solar still with the TiO_2_/carbonized OFL was 6.9 L/m^2^.day even in outdoor conditions with low solar intensities. At the same time, the TiO_2_/carbonized OFL composite achieved 98.8% COD removal efficiency and 90.8% ammonia nitrogen removal efficiency after evaporation. The excellent performance of the TiO_2_/carbonized OFL composite, particularly its high evaporation rate and pollutant removal efficiency, should motivate further research in low-cost, facile, and scalable water purification technologies. Future studies could explore the scalability of this material for large-scale water treatment applications.

## Figures and Tables

**Table 1:** Comparative analysis of solar-driven water evaporation performance among engineered materials.

Material Name	Synthesis Method	Water Evaporation Rate (kg·m^−2^·h^−1^)	Advantages	Disadvantages	Reference
PVA@PCLS	Sol-gel method	2.33	Resource recycling; high solar-to-vapor conversion efficiency	High energy consumption in the preparation process of biochar; long-term stability and durability not yet evaluated	[60]
CFC/MnO_2_/PLL	Electrostatic adsorption	2.20	Low evaporation enthalpy; enhanced light absorption and antibacterial properties	Insufficient long-term stability and durability; complex preparation methods and high costs	[51]
TiO_2_/car-bonized OFL	Hydrothermal method	2.31	High stability; efficient water purification capability	Limited applicability to pollutants	This work

## Data Availability

The original contributions presented in this study are included in the article. Further inquiries can be directed to the corresponding author.

## References

[1] Wang H.Q., Du A., Ji X.J., Zhang C., Zhou B., Zhang Z., Shen J. (2019). Enhanced Photothermal Conversion by Hot-Electron Effect in Ultrablack Carbon Aerogel for Solar Steam Generation. ACS Appl. Mater. Interfaces.

[2] Jamie B., Clarissa B., Fisher M.B., Rolf L., Rifat H., Tessa W., Gordon B. (2014). Global monitoring of water supply and sanitation: History, methods and future challenges. Int. J. Environ. Res..

[3] Lewis N.S. (2015). Introduction: Solar Energy Conversion. Chem. Rev..

[4] Liu J., Liu Q.L., Ma D.L. (2019). Simultaneously achieving thermal insulation and rapid water transport in sugarcane stems forefficient solar steam generation. J. Mater. Chem. A.

[5] Chang C., Yang C., Liu Y., Tao P., Song C., Shang W., Wu J., Deng T. (2016). Efficient Solar-Thermal Energy Harvest Driven by Interfacial Plasmonic Heating-Assisted Evaporation. ACS Appl. Mater. Interfaces.

[6] Dao V.-D., Tran C.Q., Ko S.-H., Choi H.-S. (2013). Dry plasma reduction to synthesize supported platinum nanoparticles for flexible dye-sensitized solar cells. J. Mater. Chem. A.

[7] David B., Philippe T., Abdelhamid M., Brahim L., Michel O. (2002). Photothermal imaging of nanometersized metal particles among scatterers. Science.

[8] Hessel C.M., Pattani V.P., Rasch M., Panthani M.G., Koo B., Tunnell J.W., Korgel B.A. (2011). Copper Selenide Nanocrystals for Photothermal Therapy. Nano Lett..

[9] Zhou L., Tan Y., Ji D., Zhu B., Zhang P., Xu J., Gan Q., Yu Z., Zhu J. (2016). Self-assembly of highly efficient, broadband plasmonic absorbers for solar steam generation. Sci. Adv..

[10] Zhao J., Yang Y., Yang C., Tian Y., Que W. (2018). Hydrophobic Surface Enabled Salt-blocking 2D Ti_3_C_2_ MXene Membrane for Efficient and Stable Solar Desalination. J. Mater. Chem. A.

[11] Celzard A., Pasc A., Schaefer S., Mandel K., Ballweg T., Li S., Medjahdi G., Nicolas V., Fierro V. (2019). Floating hollow carbon spheres for improved solar evaporation. Carbon.

[12] Chen T., Wang S., Wu Z., Wang X., Peng J., Wu B., Cui J., Fang X., Xie Y., Zheng N. (2018). A cake making strategy to prepare reduced graphene oxide wrapped plant fiber sponges for high-efficiency solar steam generation. J. Mater. Chem. A.

[13] Hota S.K., Diaz G. (2019). Activated carbon dispersion as absorber for solar water evaporation: A parametric analysis. Sol. Energy.

[14] Huo B., Jiang D., Cao X., Liang H., Liu Z., Li C., Liu J. (2019). N-doped graphene/carbon hybrid aerogels for efficient solar steam generation. Carbon.

[15] Miao E.-D., Ye M.-Q., Guo C.-L., Liang L., Liu Q., Rao Z.-H. (2019). Enhanced solar steam generation using carbon nanotube membrane distillation device with heat localization. Appl. Therm. Eng..

[16] Sun L., Liu J., Zhao Y., Xu J., Li Y. (2019). Highly efficient solar steam generation via mass-produced carbon nanosheet frameworks. Carbon.

[17] Surwade S.P., Smirnov S.N., Vlassiouk I.V., Unocic R.R., Veith G.M., Sheng D., Mahurin S.M. (2015). Water desalination using nanoporous single-layer graphene. Nat. Nanotechnol..

[18] Wang X., He Y., Liu X., Zhu J. (2017). Enhanced direct steam generation via a bio-inspired solar heating method using carbon nanotube films. Powder Technol..

[19] Yin Z., Wang H., Jian M., Li Y., Xia K., Zhang M., Wang C., Wang Q., Ma M., Zheng Q.-S. (2017). Extremely Black Vertically Aligned Carbon Nanotube Arrays for Solar Steam Generation. ACS Appl. Mater. Interfaces.

[20] Zhang P., Li J., Lv L., Zhao Y., Qu L. (2017). Vertically Aligned Graphene Sheets Membrane for Highly Efficient Solar Thermal Generation of Clean Water. ACS Nano.

[21] Zhu L., Gao M., Peh C.K.N., Wang X., Ho G.W. (2018). Self-Contained Monolithic Carbon Sponges for Solar-Driven Interfacial Water Evaporation Distillation and Electricity Generation. Adv. Energy Mater..

[22] Yin X., Zhang Y., Guo Q., Cai X., Xiao J., Ding Z., Yang J. (2018). Macroporous Double-Network Hydrogel for High-Efficiency Solar Steam Generation Under 1 sun Illumination. ACS Appl. Mater. Interfaces.

[23] Gao M., Peh C.K., Phan H.T., Zhu L., Ho G.W. (2018). Solar Absorber Gel: Localized Macro-Nano Heat Channeling for Efficient Plasmonic Au Nanoflowers Photothermic Vaporization and Triboelectric Generation. Adv. Energy Mater..

[24] Wang X., Sha C., Wang W., Chen Y., Yu Y., Fan D. (2019). Functionalized biomass-derived composites for solar vapor generation. Mater. Res. Express.

[25] Li J.Y., Zhou X., Mu P., Wang F., Sun H.X., Zhu Z.Q., Zhang J., Li W., Li A. (2020). Ultralight Biomass Porous Foam with Aligned Hierarchical Channels as Salt-Resistant Solar Steam Generators. ACS Appl. Mater. Interfaces.

[26] Zhou X., Li J.Y., Liu C., Wang F., Chen H., Zhao C.X., Sun H., Zhu Z. (2020). Carbonized tofu as photothermal material for highly efficient solar steam generation. Int. J. Energy Res..

[27] Xu N., Hu X., Xu W., Li X., Zhou L., Zhu S., Zhu J. (2017). Mushrooms as Efficient Solar Steam-Generation Devices. Adv. Mater..

[28] Fang J., Liu J., Gu J., Liu Q., Zhang W., Su H., Zhang D. (2018). Hierarchical Porous Carbonized Lotus Seedpods for Highly Efficient Solar Steam Generation. Chem. Mater..

[29] Fang Q., Li T., Chen Z., Lin H., Wang P., Liu F. (2019). Full biomass-derived solar stills for robust and stable evaporation to collect clean water from various water-bearing media. ACS Appl. Mater. Interfaces.

[30] Sun P., Zhang W., Zada I., Zhang Y., Gu J., Liu Q., Su H., Pantelić D., Jelenković B., Zhang D. (2020). 3D-structured carbonized sunflower heads for improved energy efficiency in solar steam generation. ACS Appl. Mater. Interfaces.

[31] Yang L., Chen G., Zhang N., Xu Y., Xu X. (2019). Sustainable biochar-based solar absorbers for high-performance solar-driven steam generation and water purification. ACS Sustain. Chem. Eng..

[32] Zhu M., Yu J., Ma C., Zhang C., Wu D., Zhu H. (2019). Carbonized daikon for high efficient solar steam generation. Sol. Energy Mater. Sol. Cells.

[33] Lu Y., Wang X., Fan D., Yang H., Xu H., Min H., Yang X. (2020). Biomass derived Janus solar evaporator for synergic water evaporation and purification. Sustain. Mater. Technol..

[34] Wilson H.M., Ahirrao D.J., Ar S.R., Jha N. (2020). Biomass-derived porous carbon for excellent low intensity solar steam generation and seawater desalination. Sol. Energy Mater. Sol. Cells.

[35] Lin Y., Zhou W., Di Y., Zhang X., Yang L., Gan Z. (2019). Low-cost carbonized kelp for highly efficient solar steam generation. AIP Adv..

[36] Long Y.J., Huang S.L., Yi H., Chen J.Q., Wu J.H., Liao Q.F., Liang H., Cui H., Ruan S., Zeng Y.-J. (2019). Carrot-inspired solar thermal evaporator. J. Mater. Chem. A.

[37] Storer D.P., Phelps J.L., Wu X., Owens G., Khan N.I., Xu H. (2020). Graphene and Rice Straw Fibre Based 3D Photothermal Aerogels for Highly Efficient Solar Evaporation. ACS Appl. Mater. Interfaces.

[38] Ma Y., Cao J. (2020). Preparation of mechanically robust Fe_3_O_4_/porous carbon/diatomite composite monolith for solar steam generation. Environ. Sci. Pollut. Res..

[39] Shan X.L., Zhao A.Q., Lin Y.W., Hu Y.J., Di Y.S., Liu C.H., Gan X. (2020). Low-Cost, Scalable, and Reusable Photothermal Layers for Highly Efficient Solar Steam Generation and Versatile Energy Conversion. Adv. Sustain. Syst..

[40] Liu J., Yang Q., Yang W., Li M., Song Y. (2013). Aquatic plant inspired hierarchical artificial leaves for highly efficient photocatalysis. J. Mater. Chem. A.

[41] Liao Y., Chen J., Zhang D., Wang X., Yuan B., Deng P., Li F., Zhang H. (2019). Lotus leaf as solar water evaporation devices. Mater. Lett..

[42] Xu K., Guo L., Ye H. (2019). A naturally optimized mass transfer process: The stomatal transpiration of plant leaves. J. Plant Physiol..

[43] Ouyang L., Zhao P., Zhou G., Zhu L., Huang Y., Zhao X., Ni G. (2018). Stand-scale transpiration of a *Eucalyptus urophylla × Eucalyptus grandis* plantation and its potential hydrological implication. Ecohydrology.

[44] Yang T., Lin H., Lin K.T., Jia B. (2020). Carbon-based absorbers for solar evaporation: Steam generation and beyond. Sustain. Mater. Technol..

[45] Zhu L., Gao M., Peh C.K.N., Ho G.W. (2019). Recent progress in solar-driven interfacial water evaporation: Advanced designs and applications. Nano Energy.

[46] Zhu L., Ding T., Gao M., Peh C.K.N., Ho G.W. (2019). Shape Conformal and Thermal Insulative Organic Solar Absorber Sponge for Photothermal Water Evaporation and Thermoelectric Power Generation. Adv. Energy Mater..

[47] Liu Y., Lou J., Ni M., Song C., Wu J., Dasgupta N.P., Tao P., Shang W., Deng T. (2015). Bioinspired bifunctional membrane for efficient clean water generation. ACS Appl. Mater. Interfaces.

[48] Huang J., He Y., Chen M., Jiang B., Huang Y. (2017). Solar evaporation enhancement by a compound film based on Au@TiO_2_ core–shell nanoparticles. Solar Energy.

[49] Sun Y., Sun S.P., Liao X.M., Wen J., Yin G.F., Pu X.M., Yao Y., Huang Z. (2018). Effect of heat treatment on surface hydrophilicity-retaining ability of titanium dioxide nanotubes. Appl. Surf. Sci..

[50] Zan G., Jiang S.W., Kim H.Y., Zhao K., Li S., Lee K., Jiang J., Kim G., Shin E., Kim W. (2024). A core–shell fiber moisture-driven electric generator enabled by synergetic complex coacervation and built-in potential. Nat. Commun..

[51] Song X., Li X., Zhu B., Sun S., Chen Z., Zhang L. (2024). MnO_2_/Poly-L-lysine co-decorated carbon fiber cloth with decreased evaporation enthalpy and enhanced photoabsorption/antibacterial performance for solar-enabled anti-fouling seawater desalination. Adv. Fiber Mater..

[52] Meng F., Wang D. (2020). Effects of vacuum freeze drying pretreatment on biomass and biochar properties. Renew. Energy.

[53] Lin T., Meng F., Zhang M., Liu Q. (2022). Effects of different low temperature pretreatments on properties of corn stover biochar for precursors of sulfonated solid acid catalysts. Bioresour. Technol..

[54] Kumar R., Strezov V., Weldekidan H., He J., Singh S., Kan T., Dastjerdi B. (2020). Lignocellulose biomass pyrolysis for bio-oil production: A review of biomass pre-treatment methods for production of drop-in fuels. Renew. Sustain. Energy Rev..

[55] Schneider J., Matsuoka M., Takeuchi M., Zhang J., Horiuchi Y., Anpo M., Bahnemann D.W. (2014). Understanding TiO_2_ photocatalysis: Mechanisms and materials. Chem. Rev..

[56] Xing L., Bao H., Gang W., Cui Z., Zhu X., Wang X. (2018). Black titania/graphene oxide nanocomposite films with excellent photothermal property for solar steam generation. J. Mater. Res..

[57] Thirugnanasambantham A., Ao Y.L., Luo Z.F., Zhang L., Li J., Denkenberger D., Wang J.Q. (2019). Energy efficient materials for solar water distillation—A review. Renew. Sustain. Energy Rev..

[58] Zhang S., Zang L., Dou T., Zou J., Zhang Y., Sun L. (2020). Willow Catkinsderived porous carbon membrane with hydrophilic property for efficient solar steam generation. ACS Omega.

[59] Li T., Fang Q., Xi X., Chen Y., Liu F. (2018). Ultra-robust carbon fibers for multi-media purification: Via solar-evaporation. J. Mater. Chem. A.

[60] Chen S., Sun L., Huang Y., Yang D., Zhou M., Zheng D. (2023). Biochar-based interfacial evaporation materials derived from lignosulfonate for efficient desalination. Carbon Neutralization.

[61] Ni A., Fu D., Lin P., Wang X., Xia Y., Han X., Zhang T. (2023). Eco-friendly photothermal hydrogel evaporator for efficient solar-driven water purification. J. Colloid Interface Sci..

[62] Jiang W., Hao S., Cang D., Ling Y., Bai X. (2008). Thermal stability of TiO_2_ nanotube array films. Chin. J. Eng..

[63] Yao J.K., Huang H.L., Xu C., Ma J., He H.B., Shao J., Jin Y.X., Zhao Y., Fan Z.X., Zhang F. (2009). Investigation on thermal stability of TiO_2_ films for application at high temperature. Surf. Eng..

